# Advancing Treatment Frontiers: Radiofrequency Ablation for Small Renal Mass—Intermediate-Term Results

**DOI:** 10.15586/jkcvhl.v10i4.303

**Published:** 2023-10-04

**Authors:** Rohit Kumar Singh, Manav Gideon, Rohan Rajendran, Georgie Mathew, Kannan Nair

**Affiliations:** 1 Department of Urology, Amrita Institute of Medical Sciences, Kochi, India;; 2 Mch Urology, Amrita Institute of Medical Sciences, Kochi, India

**Keywords:** ablation, cancer, kidney, radiofrequency, small renal mass

## Abstract

Our study aims to discern the immediate and intermediate-term oncological outcomes of the patients with small renal mass and who were surgically unfit or were having a bilateral tumor and underwent radiofrequency ablation (RFA) of the mass. We retrospectively and prospectively analyzed the status of the patients who were diagnosed to have small renal masses and were biopsy-proven renal cell carcinoma (RCC) cases, who underwent RFA at our institute from the year 2013 to 2022. Patients were followed-up for 3 years. Data regarding complications were analyzed for all patients who underwent renal RFA along with the 3-year recurrence-free survival (RFS) rate. A total of 28 patients were eligible for the study based on our inclusion and exclusion criteria. Their renal function was recorded. They underwent RFA and were followed-up for a period of 3 years for RFS. Four patients out of the total had immediate complications, out of which two developed a hematoma. Three-year-follow-ups showed six recurrences, overall having 78.6% RFS. Post-procedural renal function was stable as documented by Estimated glomerular filtration rate. Oncological results of RFA in patients with small renal masses who are surgically unfit are associated with a low risk of immediate and intermediate-term deterioration of renal function.

## Introduction

Renal cell carcinoma (RCC) is the most common renal malignancy and accounts for about 2–3% of all diagnosed adult cancers worldwide (1). In 2018, the age-standardized mortality rate was 2.9 deaths per 100,000 persons; however, from 2012 to 2016, 5-year survival increased from 51 to 79%, owing to advances in detection and management (2).

The incidence of small renal mass has increased over a couple of decades, reflecting the increase in the prevalence and detection rates. This can be attributed to increasing awareness and increased use of newer diagnostic modalities.

Traditionally radical nephrectomy, either laparoscopic or open, was considered to be the gold standard for the treatment of renal mass. This was in accordance with the oncological principles. However, this rendered the patient with a single kidney and would lead to a risk of developing chronic kidney disease (1, 3).

Due to the recent advances in surgical techniques, nephron--sparing surgeries are increasingly practiced for the management of small renal masses. Currently, there is a peaked use of nephron-sparing modalities, which includes an open, laparoscopic, and robot-assisted partial nephrectomy. Also, radical nephrectomy was considered to overtreat small renal masses. However, some patients are unfit for surgery due to their comorbidities, or some demand a no-surgical approach for small renal tumors. For these patients, advances in the ablative techniques for the treatment of small renal tumors have expanded considerably with the use of cryoablation, radiofrequency ablation (RFA), high-intensity focused ultrasound (HIFU), and microwave thermotherapy (4).

RFA is a novel minimally invasive treatment approach for the management of small renal tumors. International -studies have shown that RFA is a safe (5–8) nephron-sparing treatment (6) for small (<4 cm) (9) RCC. Due to a lack of long-term follow-up, there has been some hesitancy to use RFA as first-line management. However, recent studies have found that in properly selected patients, the outcome of RFA is comparable to that of partial nephrectomy (2). There is no data regarding the application of RFA in the Indian population. This study can provide an insight into the application of RFA in selected patients of the Indian population.

## Patients and Methods

Our study is a single institutional observational retrospective study. Data of all the included patients were collected through the health information system of our institute. All patients diagnosed to have small renal mass, i.e., size <4 cm and who were deemed unfit for surgery were included in our study. Ablation was provided by a single provider, one common consultant of the interventional radiology department of our institution. All patients underwent preprocedural biopsy and imaging to confirm the diagnosis. Biopsy was done under USG guidance using an 18G 15 cm BARD auto gun. All patients who underwent RFA between the years 2013 and 2022 were included and were followed-up for a period of 3 years.

The procedure was done under local anesthesia and the position was either prone, left, or right lateral position. It was performed under ultrasound guidance with a percutaneous approach.

The interventional radiologist inserts a thin, needle-like probe (electrode) into the kidney tissue and directly into or adjacent to the tumor. The electrode is guided with precision to ensure accurate placement. Once the electrode is properly positioned, radiofrequency energy is emitted from the tip of the electrode into the tumor tissue. This energy generates heat, which effectively destroys the tumor cells by causing coagulation necrosis essentially cooking the tumor while leaving surrounding healthy tissue relatively unharmed. Ablation was done with a 3- or 2-cm RF antenna depending on the size of the lesion. During the procedure, real-time imaging is used to monitor the location of the electrode and the extent of tissue destruction. This ensures that the entire tumor is adequately treated. After the desired level of tissue destruction is achieved, the electrode is carefully removed. Patients were kept in the recovery for 15 min and discharged the next day. The follow-up for each patient included a 6-month physical examination, sr. creatinine, chest radiography, and a contrast-enhanced computerized tomography (CT) or magnetic resonance imaging (MRI), then annually for the remaining 2 years.

### Inclusion Criteria

1. Patients who underwent RFA between 2013 and 2022 at our institute.
2. Patients who were under follow-up for 3 years post-procedure.

### Exclusion Criteria

1. Patients who were lost to follow-up.
2. Patients who did not undergo preprocedural biopsy.

### Statistical Analysis

Statistical analysis was performed using IBM SPSS version 26.0 software. Categorical variables were expressed using frequency and percentage. Numerical variables were presented using mean and standard deviation. Fisher’s exact test was used to study the statistical significance of the association of all categorical variables with recurrence. A P-value of <0.05 was considered to be significant.

## Results

Data from 2013 to 2022 were collected and a total of 36 patients were found to have undergone RFA for small renal mass. Eight patients out of 36 were lost to follow-up. Out of eight, four patients were following up with their respective physicians at their native places, and out of the remaining four, three patients followed-up for around 1 year and one patient followed up for 6 months but there was no complication noted in these patients till the time of their follow-up with us. A total of 28 patients were followed-up for a period of 3 years to analyze intermediate-term results.

**Figure 1:**
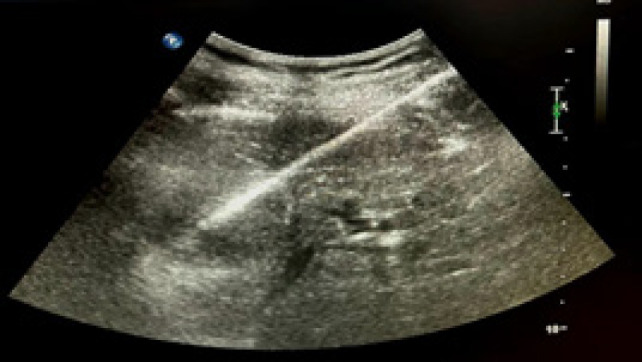
Ultrasound image of renal biopsy before ablation.

Twenty-eight patients underwent RFA for small renal masses and were followed-up for 3 years. Out of 28 patients, 20 were males (71.42%) and 8 were females (28.57%) (Table 1). The median age of the patients was 65.5 years (Range: 44). The median size of the lesions was 2.35 cm in the greatest dimension (Range: 3). The median estimated glomerular filtration rate (eGFR) before the procedure was 87.6 mL/min/1.73m^2^ (Range: 153.7) and the median eGFR after the procedure was 64.55 mL/min/1.73m^2^ (Range: 155), which did not show any significant change in renal function after ablation (P-value: 0.29). A biopsy was done before the procedure to have a histological confirmation (Figure 1). Out of 28 patients, 26 patients were found to have clear-cell carcinoma and 2 patients had papillary carcinoma of the kidney. Two patients had postoperative complications forming hematoma post-procedure, which was managed by tight compression over the skin and oral antibiotics. There was no other associated complication or toxicity. Two patients out of 28 had VHL association. The mean time of the procedure was 10.07 min. The mean duration of stay in the hospital was 1 day.

**Table 1:** Distribution of patients according to gender, recurrence at 3 years, and location of recurrence.

Gender	Number (*n*)		Percentage
Male	20		71.42
Female	8		28.57
Location	No recurrence	Recurrence	Total (***n***)
Lower pole	6 (100%)	0 (0%)	6
Mid pole	8 (80%)	2 (20%)	10
Upper and middle pole	0 (0%)	2 (100%)	2
Upper pole	8 (80%)	2 (20%)	10

Recurrence was noted in six patients during 3 years of follow-up. Four patients had recurrence during the first year of follow-up and two patients had recurrence during the third year of follow-up. All patients who had recurrence were biopsy-proven cases of clear-cell carcinoma. No recurrence was noted in the papillary carcinoma group. Recurrence was noted in two lesions in the mid pole, two lesions in the upper and mid pole, and two lesions in the upper pole (Table 1). On analysis, there was no statistically significant association of recurrence with size, gender, type of RCC, and location of lesion. Patients with recurrence were subjected to re-ablation and are under follow-up.

## Discussion

**Figure 2:**
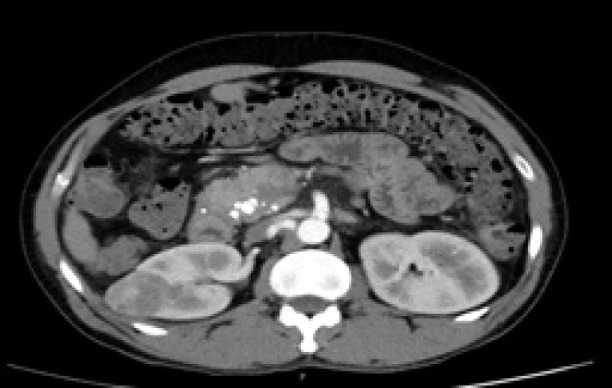
Pre-ablation computerized tomography (CT) scan showing a small renal mass in the mid-pole of the right side kidney.

**Figure 3:**
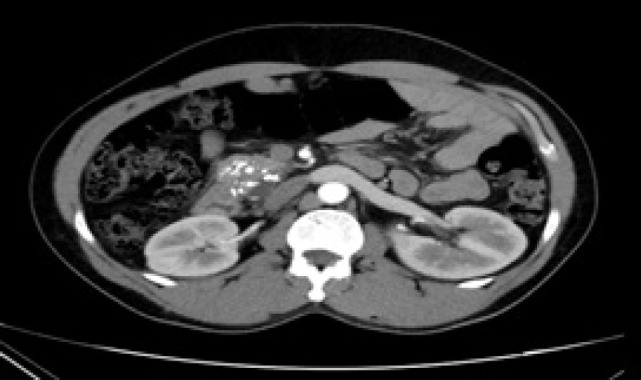
Computerized tomography (CT) scan after 3-year follow-up showing almost complete resolution of the mid-polar small renal mass in the right kidney.

The growing issue of managing numerous small kidney masses, which exhibit unpredictable progression, within a population of uncertain lifespan, presents a challenging situation for medical professionals. This scenario has prompted the development of ablative methods. While the preferred approach, partial nephrectomy, may not be suitable for individuals with significant underlying health conditions and a high risk of anesthesia-related complications, ablative techniques have become noteworthy. These techniques offer several benefits, including the ability to be performed in an outpatient setting without the need for general anesthesia, which ultimately reduces the potential for complications. In our study, we followed the patients for the intermediate-term results post-ablation that is after a period of 3 years. Most of our patients were seen to have achieved almost complete resolution at the intermediate term on CT imaging (Figures 2 and 3). Our study showed that the mean age of patients undergoing ablation was 63.35 years. In a study by Johnson DB et al., the average patient age was 61.2 years (10). The majority of the patients had clear-cell RCC on histology (92.85%) and two patients had papillary carcinoma (7.14%). In a study done by Psutka SP et al., pathologic analysis of renal biopsies demonstrated that tumors were of the clear cell variant in 97 (54.1%), papillary in 33 (17.4%), chromophobe in 4 (2.2%), an oncocytic variant of RCC in 5 (2.7%), and RCC, not otherwise specified histologic type, in 46 (24.9%) (11). In our study, there was no significant effect on renal function pre- and post-ablation. In a study done by David Curry et al., 19 (24%) patients had a subsequent deterioration in eGFR with a median change of 7 (IQR [interquartile range]: 5–11). Renal function deterioration was seen significantly more frequently and at a significantly greater level in the cohort of patients with pre-existing renal impairment (P < 0.005/P = 0.001). No significant difference in the number affected or level of deterioration was seen for gender (P = 0.1/P = 0.069), diabetes (P = 1.0/P > 0.99), hypertension (P = 0.296/P = 0.213), or vascular disease (P = 1.0/P = 0.14) (4). In a study by Lucas SM et al., a GFR decrease of less than 45 mL per minute per 1.73 m^2^ was noted in 3 of 63 patients in the RFA group, 3 of 55 in the partial nephrectomy group, and 11 out of 40 in the radical nephrectomy group (P 0.001) (12).

Our study showed that 2 out of 28 developed hematoma post-ablation but both were observed in the ward overnight and were discharged the next day. The review article by Kurup AN et al. states that complications following renal ablation are seen infrequently. A multi-institutional review of 271 RFA and cryoablation procedures demonstrated an overall complication rate of 11% (13). However, the inclusion of both intraoperative and percutaneous treatments in this review confounds the interpretation of the outcomes. A meta-analysis comparing percutaneous and surgical renal ablation procedures found a significantly lower major complication rate of 3.1% for percutaneous ablation versus 7.4% for surgical cases (14). A review of several large series of percutaneous renal RFA from the literature reveals an overall complication rate of 8 to 13% with major complication rates of 4 to 6% (15) (Table 2) The mean time of the procedure was 10.07 min without any major bleeding. The mean duration of stay in the hospital was 1 day. A study by Hwang et al. showed that the mean operative time and intraoperative blood loss were 243 ± 29 min and 67 ± 9 cc, respectively, and the average postoperative stay was 2.9 days (16). In our study, recurrence was noted in six patients (21.4%) during 3 years of follow-up. The calculated recurrence-free survival (RFS) was 78.6%. Four patients (66.7%) had recurrence during the first year of follow-up and two patients (33.33%) had recurrence during the third year of follow-up. All patients who had recurrence were biopsy-proven cases of clear-cell carcinoma (100%). No recurrence was noted in the papillary carcinoma group. Recurrence was noted in two lesions in the mid pole (20%), two lesions in the upper and mid pole (100%), and two lesions in the upper pole (20%). In a study by David Curry et al., residual tumor was present in 14 (18%) patients on initial imaging with 10 of these proceeding to successfully salvage RFA. Tumor size was not a predictor of residual disease (P = 0.49). Tumor recurrence was seen in four (5%) patients with a median time to recurrence of 15 months (IQR 6.25–33.75 months) (4). In a study by Hwang et al., of the 24 tumors treated, only 1 (4%) exhibited contrast enhancement (HU change greater than 10) and met radiographic criteria for tumor recurrence (16). Olweny et al. did a study of 142 patients. Univariable and multivariable analyses revealed that age, tumor size, duration of follow-up, histology (clear cell vs non–clear cell), and approach (RFA vs PN) were not significant predictors of any of the oncologic outcomes (7). Our study also does not show any significant association of recurrence with size, gender, type of RCC, or location of lesion.

**Table 2:** Illustration of previous studies on radiofrequency ablation of renal masses.

Study	Study design	Participants	Intervention	Reported outcomes
Johnson DB et al.	Retrospective	25	RFA	Good oncological outcomes with no effect on renal function
Psutka SP et al.	Retrospective	185	RFA	Low rate of recurrence
David Curry et al.	Retrospective	89	RFA	Durable oncological outcomes, low risk of complications, minimal effect on renal function
Lucas SM et al.	Retrospective	242	RFA vs partial nephrectomy vs radical nephrectomy	Preserved renal function by nephron-sparing surgeries
J J Hwang et al.	Retrospective	17	RFA	Superior outcomes of 200 watt power of RFA as compared to low wattage
Ephrem O. Olweny et al.	Retrospective	74	RFA vs partial nephrectomy	Improved and comparable long-term oncological outcomes

## Limitations of the Study

Our study’s sample size might not be representative of the entire population or might not have sufficient statistical power to detect subtle effects. The fact that over 20% of the patients were lost to follow-up is a significant concern. This loss could introduce bias, as patients who dropped out of the study might have different outcomes or characteristics compared to those who remained. This could impact the reliability and validity of the study’s conclusions. Lastly, the retrospective nature of our study means that data introduce the potential for selection bias.

## Conclusion

RFA stands as a secure and efficient choice for addressing small renal masses in patients for whom surgery is not suitable. This procedure is time-efficient and doesn’t necessitate extensive postoperative monitoring. RFA has the potential to serve as an effective treatment for individuals dealing with small renal masses, especially those who are unsuitable candidates for surgery due to serious medical conditions or chronic kidney disease with diminished renal function. Appropriate post-ablation follow-up is imperative, demanding patients’ diligent adherence to scheduled outpatient visits, as recurrence might necessitate repeat ablation.
